# An Exploratory Study of Educators’ Perspectives Towards Hospital School Program Support for Children with Special Health Care Needs After Hospitalization

**DOI:** 10.5334/cie.110

**Published:** 2024-03-15

**Authors:** Heather E. Ormiston, Polly R. Husmann, Kristin C. Wikel, Chelsey Ruark, Debra L. Reisinger, Michelle J. Curtin

**Affiliations:** 1 Indiana University Bloomington, US; 2 Indiana University School of Medicine, US; 3 Ball State University Teachers College, US; 4 Cincinnati Children’s Hospital Medical Center, University of Cincinnati College of Medicine, US; 5 Wake Forest School of Medicine, US

**Keywords:** inpatient hospitalization, hospital school program, educator, survey, student

## Abstract

More than 14 million children in the United States are identified as children with special healthcare needs (CSHCN). Rates of hospitalization for CSHCN with chronic conditions as well as re-admissions have been increasing in recent years. For hospitalized children transitioning back to their school of record, a host of issues may arise such as socioemotional concerns, peer rejection, and being behind in academics. Hospital-based school programs (HBSPs) play an important role in the transition back to a child’s school of record. Utilizing a database of inpatient CSHCN at a midwestern children’s hospital’s HBSP, private and public-school educators associated with the previously hospitalized CSHCN were asked to complete an online survey to gather their perspectives related to the child’s transition back to the school of record upon hospital discharge. Overall, educators’ perspectives of the HBSP were positive while perceptions related to communication provided by the HBSP were mixed. Educators surveyed reported a lack of training related to working with CSHCN. Finally, accommodations and services offered to students upon return to school focused mostly on academic performance and attendance. Study limitations and implications for practice in schools are discussed.

More than 14 million children in the United States are identified as children with special healthcare needs (CSHCN; Maternal and Child Health Bureau [MCHB], 2022). CSHCN are defined as “those who have or are at increased risk for a chronic physical, developmental, behavioral, or emotional condition and who also require health and related services of a type or amount beyond that required by children generally” (MCHB, 2022, p. 1). Chronic conditions are disorders, disabilities, or diseases that interfere with daily functioning for at least three months, involve physical and/or cognitive impairment, and require frequent medical intervention including hospitalizations (Perfect & Moore, 2019). CSHCN encompass children with significant behavioral health needs (e.g., conduct problems, depression, anxiety) as well as physical health/medical needs (e.g., asthma, heart condition, allergies; Ghandour et al., 2022). Prevalence rates vary, with recent estimates indicating approximately 15–25% of children (under the age of 18) receive ongoing medical care due to a chronic illness on an annual basis (Berger et al., 2018; Canter & Roberts, 2012; Ghandour et al., 2022; Perfect & Moore, 2019). For children older than 10, behavioral health conditions were among four of the top 10 most common diagnoses associated with inpatient stays (Weiss et al., 2022). Overall, rates of hospitalization for CSHCN with chronic conditions as well as inpatient re-admissions have been increasing (Bucholz et al., 2019).

While the length of pediatric hospital stays depends on the child’s needs and diagnosis, the “average time students with multiple disabilities are absent from school is 28.9 days each time they are hospitalized” (Boff et al., 2021, p. e75). Academic outcomes demonstrate increased special education identification and lower educational attainment overall for this population (Champaloux & Young, 2015; Lum et al., 2017). Greater disease severity, including illnesses and/or treatments that affect cognitive functioning, has been found to negatively correlate with academic performance, absenteeism, and grade retention (Lum et al., 2017).

Given advances in medical care, CSHCN are more often able to survive acute and chronic illnesses and integrate back into the school environment following a hospitalization (Colbert et al., 2020; Irwin et al., 2018). However, a host of issues arises as children transition from inpatient hospitalization back to school. This includes socioemotional concerns in children, such as anxiety or depression (Fremont, 2019; Magalhães et al., 2018; Savina et al., 2014; Shaw & McCabe, 2008), peer rejection (Helms et al., 2016; Preyde et al., 2018; Shaw & McCabe, 2008), being behind in academics (Magalhães et al., 2018; Preyde et al., 2018; West et al., 2013), and medication and treatment side effects (Clemens et al., 2011; Colbert et al., 2020), such as lethargy and cognitive dulling. Further, the needs of CSHCN vary dramatically based upon the nature of the hospitalization with children in some studies reporting struggles to navigate the social and academic complexities of the school environment, while also feeling little support from school staff (Clemens et al., 2011; Magalhães et al., 2018; Savina et al., 2014; White et al., 2017).

## Educator Preparedness

A vast majority of educators (e.g., teachers, school counselors, administrators) have a history of teaching or interacting with a child with a chronic health condition and play an important role in their care (Barnard-Brak et al., 2017). However, educators report having little knowledge of how to educationally support students with chronic illness in a general education setting (Colbert et al., 2020; Hinton & Kirk, 2015; Irwin et al., 2018), while increasingly bearing the burden of caring for the medical needs of students given the lack of nurses and health aides in schools (West et al., 2013). The limited training and preparation on chronic health conditions for educators compromises the successful inclusion of these children in the school setting (Legislative Agency for Students with Health Conditions [LASHC], 2017). Further, there is often a lack of coordination between hospitals and schools upon students’ reentry from hospitalization (Barnard-Brak et al., 2017; Magalhães et al., 2018; White et al., 2017). School-based factors related to successful reentry include coordination among school personnel, support and understanding in schools, and consistent follow-through (Clemens et al., 2011; Marraccini & Pittleman, 2022).

As such, there has been a recent push to provide educators with recommendations and considerations for supporting students upon reentry from hospitalization (e.g., Savina et al., 2014; Shaw et al., 2010). One way to increase educators’ knowledge is through specific trainings on supporting students with chronic illnesses. The success of these students often relies on educators and related school personnel being properly trained in understanding and supporting children with these illnesses (Clemens et al., 2011), and educators endorse a desire to receive training in this area (Irwin et al., 2018).

## Hospital-Based School Programs

Seen as the preferred model in which to deliver educational services to hospitalized children (Boff et al., 2021), hospital-based school programs (HBSPs) play a critical role in the delivery of educational services to children (Boff et al., 2021; Ratnapalan et al., 2009; Rodriguez et al., 2024; Steinke et al., 2016) via their own hospital-employed educators (Boff et al., 2021). “[H]ospitals are increasingly recognizing the importance of an H[B]SP as a core component of each child’s hospitalization” (Steinke et al., 2016, p. 31). Additionally, access to a teacher and continuing with school, even while hospitalized, provides children with a sense of routine (Magalhães et al., 2018; Ratnapalan et al., 2009) and attempts to offer hospitalized children the same educational opportunities as their typically developing peers (Caggiano et al., 2021). HBSP teachers differ considerably from “traditional” school-based teachers in that they are often teaching students one-on-one, across multiple grade levels over the course of an entire day (Steinke et al., 2016).

HBSP services are meant to mirror, to the extent possible, the instruction students would receive if they were in the “typical” classroom and should commence as soon as possible for the hospitalized patient (Committee on School Health, 2000; Magalhães et al., 2018). With parental consent obtained, communication with the student’s school of record is an important role of the HBSP teacher in order to acquire information about the child’s skills and current educational programming (Caggiano et al., 2021). HBSP teachers may communicate with general education teachers, school nurses, school-based mental health personnel (e.g., school psychologists, school counselors), administrators, and parents regarding the most appropriate educational programming for the child (Committee on School Health, 2000). Such communication is particularly important for children with chronic conditions that require frequent or intermittent hospitalization (Committee on School Health, 2000).

### The Current Study’s Hospital-Based School Program

The HBSP of focus in this study is based in a free-standing, 354-bed children’s hospital in the midwestern United States. The hospital is funded predominantly through state grants and donations. Based on the hospital’s 2020 financial reports, it had 17,960 admissions and observation stays that year. The hospital has 456 inpatient beds across medical services and is the only Emergency Department Behavioral Health Access Center for the state. It serves more than 1,500 inpatient students per year, supporting children from nearly 90% of the counties across the state. Approximately 25% of the HBSP’s patients identify as non-White. Hundreds of students are from rural areas with limited access to medically related resources (e.g., outpatient clinics, full service hospitals).

During the academic year during which the study was conducted, the school program was staffed by seven full-time and four-part time licensed teachers, one full-time school manager who is also a licensed teacher, and one part-time instructional assistant who provide services to inpatient students. The HBSP serves three primary types of students with medical needs: hospitalized students who are medically stable as defined by the medical team; partially hospitalized students receiving care for eating disorders; and students in select outpatient clinics (i.e., cancer, blood disorders, cystic fibrosis, during dialysis/after kidney transplant, and traumatic brain injury). These specific outpatient clinics have teacher support due to the increased complexity of the medical needs. Due to constantly changing rates of hospitalized youth at a given time, exact student-teacher ratios cannot be calculated.

All hospitalized students receive an initial consult with an HBSP teacher on the first school day (i.e., Monday through Friday) of their admission. Those with limited duration stays, when asked not to consult by the medical team, or those with medical status of critical or unstable do not receive a consult. In the outpatient setting, all students receiving dialysis have an initial consultation at their first treatment session and meet with a teacher every return session. Students attending the other outpatient clinics with teacher support connect to the school program via care transfer from the inpatient school liaison at the time of student discharge from the hospital. The HBSP runs according to the state’s academic calendar, except for students in the three areas of inpatient behavioral health, partial hospitalization for eating disorders, and inpatient rehabilitation (these units have more staffing and services, are open year-round, and have no extended summer break), and is closed for federally recognized holidays.

The primary services provided by the HBSP are broadly broken down into (a) direct instruction, or teaching; and (b) advocacy services. During direct instruction, students receive educational intervention as dictated by the school of record while they are receiving medical services. Direct instruction is carried out in small-group classes (e.g., inpatient psychiatric hospitalized students, partial hospitalization for eating disorders, inpatient cancer center) or on an individual basis while medically stable for hospitalized patients and individuals receiving dialysis due to the intensity of dialysis treatment as tolerated by the student. Other than students going to and from dialysis, and students at the partial hospitalization program for eating disorders, no outpatient student receives school program direction instruction.

Advocacy services encompass a wide array of supports. This includes (a) education to parent/guardian, school personnel, administrator(s), and/or peers regarding the child’s medical and educational needs; and (b) engagement in school meetings for re-entry, such as advocating for Individualized Education Programs (IEPs) for students eligible for special education services or 504 Plan meetings for students needing educational accommodations for medical reasons. Advocacy has also led to development of multiple materials to support collaboration between families and schools including transition materials, special education resources, and forms for a variety of needs (e.g., medical homebound forms).

## Purpose

While the importance of supporting students with chronic illness upon their re-entry into their school of record has been noted, there is a scarcity of literature examining students’ transitions from inpatient hospitalizations to school settings (Blizzard et al., 2016; Canter & Roberts, 2012). For example, although some literature exists highlighting the role of support personnel such as special education teachers (Simon & Savina, 2010), school psychologists (Marraccini & Pittleman, 2022), and school counselors (Vanderburg et al., 2023), this remains an area ripe for exploration (Barraclough & Machek, 2010; Hinton & Kirk, 2015; Rodriguez et al., 2024). There is also a lack of research focusing on the role the hospital plays in transitioning students back to school and a limited understanding of the perspectives of school of record educators during this transition (Rodriguez et al., 2024). As a result, additional exploration of how hospital school programs and educators can support this population following inpatient hospitalization is encouraged (Barraclough & Machek, 2010; Boff et al., 2021; Hinton & Kirk, 2015; Rodriguez et al., 2024), including a documented need to examine HBSP services through the lens of “educators from patients’ schools of record” (Steinke et al., 2016, p. 42).

The purpose of the current study was to examine educators’ perspectives regarding the services of a HBSP in supporting children transitioning from inpatient hospitalization back to their school of record. The following research questions were examined:

1. What are school personnel’s perceptions of the quality of school reintegration services from the HBSP following inpatient hospitalization?
2. What is school personnel’s training, knowledge of, and experience with children with chronic illness?
3. What perceived support did school personnel receive from the HBSP as the student returned to school?
4. What do school personnel find valuable regarding school programming provided by the HBSP?
5. What more do school personnel feel the HBSP should be doing to provide top quality services to hospitalized patients to support transition back to school?

## Method

### Procedures

To answer the research questions, the study sought to examine the perspectives of private and public school educators who had a student admitted to a midwestern children’s hospital and received services from the HBSP at some point during the 2021–2022 school year. The HBSP gathered contact information on school personnel associated with the hospitalized children at initial consult. Upon university Institutional Review Board (IRB) approval, an HBSP staff member shared the contact information (e.g., email addresses) of those school personnel with the first and second authors. Educators whose students had received services from the HBSP at any point during the 2021–22 academic year were emailed a link to complete an online Qualtrics (2018) survey comprised of items from measures outlined below, and included forced-choice, Likert, multiple-choice, and open-ended items. Two reminder emails were sent, and the survey was open for approximately three weeks. Participants were eligible to receive a small payment (i.e., Amazon gift card) upon completion of data collection.

### Sample Demographics

School of record personnel completed self-demographic items related to gender identification, years of teaching experience, highest degree earned, area of certification (e.g., general education, special education, administrator), and grade level(s) taught (see Table 1). A total of 39 survey responses were analyzed after the data were cleaned. Results indicated that survey respondents are predominantly female (80%) and identified as white (92.5%). Respondents also indicated that they were predominantly from urban (45%) or suburban (30%) settings and 75% had a master’s degree or equivalent. For number of years as an educator, the most common response was 21 or more years (40%) followed by 11–15 years (22.5%). Overall, 75% indicated that they had more than 10 years’ experience. Based on State Department of Education demographics, the sample had more years’ experience in education than is typical for educators within the state (55.7% of state educators have more than 10 years’ experience; Indiana Department of Education, 2023). Finally, although over 90% of surveys were emailed to public school educators, the survey did not collect specific data related to school type (e.g., public school, private school, charter school) by respondent.

**Table 1:** Survey Participant Demographics (N = 40).


*DEMOGRAPHIC* / CHARACTERISTIC	COUNT (%)

*Level* (select all that apply)	

Preschool	5 (12.5%)

Primary	15 (37.5)

Intermediate	13 32.5)

Middle school/Jr. high	16 (40)

High school	20 (50)

*School Locale*	

Rural	7 (17.5)

Town	3 (7.5)

Suburban	12 (30)

Urban	18 (45)

*Professional Role*	

School counselor	15 (37.5)

Classroom teacher	8 (20)

Administrator	7 (17.5)

Special education	5 (12.5)

Social worker	2 (5)

Health aide/nurse	2 (5)

*Sex*	

Woman	32 (80.0)

Man	6 (15.0)

Non-binary	1 (5.0)

Did not respond	1 (5.0)

*Race/Ethnicity*	

Black (African or African American)	2 (5)

White (Caucasian)	37 (92.5)

Multiple races	1 (2.5)

*Years Working in Education*	

1–3	2 (5)

4–6	2 (5)

7–10	6 (15)

11–15	9 (22.5)

16–20	5 (12.5)

21+	16 (40)

*Highest Degree Earned*	

Bachelor’s	9 (22.5)

Master’s	30 (75.0)

Did not respond	1 (2.5)


### Measures

The online survey was adapted from two existing measures, the School Reintegration Questionnaire (SRQ; Marraccini et al., 2019) and the School Health Questionnaire (SHQ; Clay et al., 2004). Items in the survey were also loosely based on past studies of similar topics (Blizzard et al., 2016; Clay et al., 2004; Vickers & Romero, 2021). Specifically, seven items from the SRQ were either directly incorporated (e.g., “Which of the following are typically included in your school’s re-entry plans for students returning to school following hospitalization?”) or adapted into the current survey. For instance, the SRQ has separate items asking participants about accommodations for various academically oriented tasks such as for class attendance, assignments, and test taking. To simplify, we combined these items into one question. Four items from the SHQ were adapted into questions on the current survey. Specifically, items such as “On a scale of 1 to 4, with 1 being not comfortable at all and 4 being very comfortable, please rate your comfort level in having a student with a chronic illness in your classroom/school” and “Where do you get most of your information about pediatric chronic illnesses?” were directly modeled off items from the SHQ. Open-ended items were developed directly in collaboration with the researchers and the school personnel at the HBSP.

Some open-ended items were meant to expand upon previous survey responses as modeled in the SRQ and SHQ. For instance, if a respondent selected “other” for the item asking about the types of services the HBSP helped develop for students returning to school following hospitalization, respondents were prompted to “please describe ‘other.’” Other open-ended items were designed specifically to capture perspectives of participants not otherwise included in the survey items specifically related to HBSP services more broadly from a program evaluation perspective. It should be noted that psychometric properties of the original measures (i.e., the SRQ and SHQ) are not available in published literature.

The final survey consisted of 21 content items and eight demographic items. Content items included multiple-choice/forced-choice items related to satisfaction of HBSP services, the types of services offered by the HBSP, the types of services offered in student reentry plans, personnel involved in reentry plans, and procedures for reentry the HBSP should offer. Open-ended response items were also included on items asking about satisfaction of HBSP services (e.g., “What is your reasoning for your answer to Question 5?”) and the types of services offered by the HBSP not previously listed in the survey item. Finally, two open-ended questions asked, “What has been the most helpful thing(s) about the services you and/or your student received from [redacted]?” and “What would improve the services provided by the [redacted]?”

Survey items were piloted with three teachers—one general education teachers and two special education teachers—at the elementary and secondary levels across the state. Adjustments to the wording of some items were made based on their feedback. A full copy of the survey is available from the first author upon request.

### Data Analysis

A total of 291 surveys were initially sent. After accounting for and attempting to rectify emails that were undeliverable/no longer valid, a total of 199 emails were successfully sent. The final sample yielded 39 respondents who completed some portion of the survey and were included for analysis, yielding a 19.6% response rate. All data from the survey was downloaded into an Excel file, which was used to clean the data from partial responses that were deemed unusable.

Cronbach’s alpha on the non-demographic questions indicated a good degree of reliability (α = 0.88). Correlations were then run between the services and accommodations used and the level of satisfaction with the program. Correlations were also run between the services and accommodations used and the respondents’ years of experience to determine if participants became more aware of additional services with additional years of service. All *p* cut-off values were set to 0.05. Average numbers of accommodations and services utilized were then analyzed by locale (e.g., urban, suburban, rural), and descriptive data for quality of individual services were also examined.

The open-ended questions were also examined to determine the most common responses. Familiarity was established by reading through all of the responses several times. An inductive thematic analysis model (Braun & Clark, 2006) was used to assign codes to the responses and then condensed into overall themes by the second author. A single author identified the initial codes and themes to avoid issues with inter-rater reliability. However, the first two authors discussed and agreed upon the ultimate interpretation for broader dependability. This interpretation was then examined with the quantitative analyses described above to triangulate a more complete picture of the educators’ opinions and attitudes toward the school program services.

## Results

### Individuals Involved in Services

First, to understand the elements of school re-entry plans currently in place, we asked participants about the individuals and services involved in their schools. Consistent with items modeled from the SRQ, response options included *mandatory, optional, not available*, and *unsure/unknown*. Most commonly, the student and their family were *almost always* involved in re-entry planning (79.3% and 90.3%, respectively; see Table 2). School counselors (77.8%) and general education teachers (54.8%) were also *almost always* involved, whereas the following individuals were *sometimes* involved: assistant principals (62.1%), school psychologists (58.3%), special education teachers (48.3%), and school nurses (48.1%). Ten respondents (32.2%) indicated principals were *never* involved while eight respondents (33.3%) indicated school psychologists were *never* involved.

**Table 2:** How Often Was Each of the Following Individuals Involved in Any Process Related to Students Returning to School Following Hospitalization While Working with the [Hospital] School Program?


ROLE	ALMOST ALWAYS COUNT (%)	SOMETIMES COUNT (%)	NEVER COUNT (%)

Parents/family	28 (90.3%)	2 (6.4%)	1 (3.2%)

Student	23 (79.3)	6 (20.7)	0 (0)

School counselor	21 (77.8)	4 (14.8)	2 (7.4)

General education teacher	17 (54.8)	10 (32.2)	4 (12.9)

Special education teacher	13 (44.8)	14 (48.3)	2 (6.9)

School nurse	12 (44.4)	13 (48.1)	2 (7.4)

Principal	10 (32.2)	11 (35.5)	10 (32.2)

Vice/assistant principal	7 (24.1)	18 (62.1)	4 (13.8)

School social worker	7 (30.4)	12 (52.2)	4 (17.4)

School psychologist	2 (8.3)	14 (58.3)	8 (33.3)


*Note*. Frequency count. Response rates varied (*N* = 23–31).

### Elements Included in Re-Entry Plans

In terms of elements involved in a typical re-entry plan, participants rated the items from their perspective of what was typical “standard operating procedure” for their school. Consideration of hospital recommendations was most commonly *mandatory* (63.9%), while consideration of previous school-based evaluations (41.7%) and recommendations by outside mental health professionals (36.1%) and family members (33.3%) were also endorsed as being *mandatory*. A vast majority of other re-entry plan elements were considered *optional* (e.g., recommendations provided by the student, a gradual return to school using transition space outside of school). For each response option, a notable number of respondents were *unsure* of what elements were available as part of a typical re-entry plan. See Table 3 for a summary of elements typically included in re-entry plans.

**Table 3:** Which of the Following Are Typically Included in Your School’s Re-Entry Plans for Students Returning to School Following Hospitalization?


RE-ENTRY PLAN ELEMENT	MANDATORY COUNT (%)	OPTIONAL COUNT (%)	NOT AVAILABLE COUNT (%)	UNSURE/UNKNOWN COUNT (%)

Consideration of hospital evaluations/recommendations	23 (63.9%)	6 (16.7%)	0 (0.0%)	7 (19.4%)

Consideration of previous school-based evaluations	15 (41.7)	10 (27.8)	1 (2.8)	10 (27.8)

Recommendations provided by outside mental health professionals	13 (36.1)	14 (38.9)	0 (0.0)	9 (25.0)

Recommendations provided by parents/other family members	12 (33.3)	17 (47.2)	0 (0.0)	7 (19.4)

Recommendations provided by school counselors	10 (27.8)	17 (47.2)	2 (5.6)	7 (19.4)

Recommendations provided by school psychologists	9 (25.0)	17 (47.2)	3 (8.3)	7 (19.4)

Recommendations provided by school social workers	9 (25.0)	16 (44.4)	4 (11.1)	7 (19.4)

Recommendations provided by teachers	9 (25.0)	20 (55.6)	0 (0.0)	7 (19.4)

Recommendations provided by student	6 (16.7)	23 (63.9)	0 (0.0)	7 (19.4)

Separate school for transition prior to return to classes	1 (2.8)	13 (36.1)	15 (41.7)	7 (19.4)

Gradual return to school using transition space outside of school (e.g., separate facility for academic and social-emotional support; home-bound educational services)	1 (2.8)	20 (55.6)	6 (16.7)	9 (25.0)

Gradual return to academic classes using transition space within school (e.g., separate area for academic and social-emotional support)	1 (2.8)	23 (63.9)	4 (11.1)	8 (22.2)

Recommendations provided by others	0 (0.0)	21 (58.3)	3 (8.3)	12 (33.3)

Gradual return to school using another type of service	0 (0.0)	6 (16.7)	4 (11.1)	26 (72.2)


*Note*. Frequency count (*N* = 36).

### Available Services and Accommodations

Next, respondents indicated which services and accommodations the HBSP helped to develop for their student’s re-entry. Response options were *available* and *not available*. In terms of services the HBSP helps develop and the school of record provides, support with time management and assignment make-up was most commonly endorsed as *available* (51.5%), with students checking in regularly with an adult endorsed by 45.5% of participants (see Table 4). Peer and adult mentoring were most commonly endorsed as *not available* (90.9%) while mental health supports, such as individual (60.6%) and group counseling (72.7%) were also commonly *not available*. Additionally, supports such as social skills groups (75.8%), or a transition space within (60.6%) or outside of school (75.8%), were also typically *not available*. In terms of accommodations, those most directly related to student attendance and academic performance were often rated as *available* (see Table 5). For instance, excused absences following the return to school (92.8%), extended deadlines for assignments (85.7%), missing work forgiveness (85.7%), and reduced assignments (82.1%) were commonly endorsed as *available*. Interestingly, nurse visits were the accommodation most often listed as *not available* (82.1%).

**Table 4:** Which of the Following Services Did the [Hospital] School Program Help Develop for Students Returning to School Following Hospitalization?


SERVICE	AVAILABLE COUNT (%)	NOT AVAILABLE COUNT (%)

Support with time management/assignment make up	17 (51.5%)	16 (48.5%)

Student checks in regularly with an adult	15 (45.5)	18 (54.5)

Transition space within school (e.g., separate area for academic or social-emotional support)	13 (39.4)	20 (60.6)

Individual counseling	13 (39.4)	20 (60.6)

Early outreach (e.g., personalized outreach for students with high absence rates)	12 (36.4)	21 (63.6)

Self-monitoring instruction	11 (33.3)	22 (66.7)

Tutoring	9 (27.3)	24 (72.7)

Group counseling	9 (27.3)	24 (72.7)

Transition space outside of school (e.g., separate facility for academic or social-emotional support)	8 (24.2)	25 (75.8)

Social skills group	8 (24.2)	25 (75.8)

Peer mentoring program	3 (9.1)	30 (90.9)

Adult mentoring program	3 (9.1)	30 (90.9)


*Note*. Frequency count (*N* = 33).

**Table 5:** Which of the Following Accommodations Did the [Hospital] School Program Help Implement for Students Returning to School Following Hospitalization?


ACCOMMODATION	AVAILABLE COUNT (%)	NOT AVAILABLE COUNT (%)

Excused absences following return to school	26 (92.8%)	2 (7.1%)

Extended deadlines for assignments	24 (85.7)	4 (14.3)

Missing work forgiveness	24 (85.7)	4 (14.3)

Reduced assignments/workload	23 (82.1)	5 (17.8)

Extended time limits for tests	21 (75.0)	7 (25.0)

Opportunity to take tests in quiet location	21 (75.0)	7 (25.0)

Breaking long tests into shorter time blocks	20 (71.4)	8 (28.6)

Alternatives to traditional testing (oral presentation, projects, etc.)	19 (67.8)	9 (32.1)

Health plan	19 (67.8)	9 (32.1)

Opportunity to retake tests	16 (57.1)	12 (42.9)

Pass to attend school late or leave school early	16 (57.1)	12 (42.9)

Open-book tests	13 (46.4)	15 (53.6)

Universal pass (to visit school counselor, social worker, etc., at any time)	13 (46.4)	15 (53.6)

Emergency response/evaluation plan	13 (46.4)	15 (53.6)

Medication administration	12 (42.9)	16 (57.1)

Special dietary plan	11 (39.3)	17 (60.7)

Nurse visits	5 (17.8)	23 (82.1)


*Note*. Frequency count (*N* = 28).

### Procedures Provided by HBSP

Respondents were then asked to indicate which services they would find most helpful for HBSP to provide (see Table 6). Most participants endorsed that holding meetings with families about re-entry needs (70.6%), developing individualized re-entry plans (61.8%), meeting with the student about their re-entry needs (55.9%), and phone communication between the school of record and hospital staff (44.1%) should be *mandatory* services offered by the HBSP. Most other services, with the exception of in-person visits by school staff to the hospital (20.6% endorsed *not needed*), were endorsed most frequently as *optional* services the HBSP should provide. Via open-ended responses, participants indicated using access to online/hybrid options and utilizing partial or shortened days as additional services for a student’s gradual return to school.

**Table 6:** Which of the Following Procedures Should the [Hospital] School Program Provide to Schools for Students Returning to School Following Hospitalization?


RE-ENTRY PLAN ELEMENT	MANDATORY COUNT (%)	OPTIONAL COUNT (%)	NOT NEEDED COUNT (%)	UNSURE/UNKNOWN COUNT (%)

Meeting with family about re-entry needs	24 (70.6%)	8 (23.5%)	0 (0.0%)	2 (5.9%)

Development of an individualized re-entry plan	21 (61.8)	9 (26.5)	0 (0.0)	4 (11.8)

Meeting with student about re-entry needs	19 (55.9)	13 (38.2)	0 (0.0)	2 (5.9)

Phone communication with hospital staff	15 (44.1)	16 (47.1)	0 (0.0)	3 (8.8)

Referral for special education/504 evaluation	8 (23.5)	23 (67.6)	1 (2.9)	2 (5.9)

In-person visits by school staff to the hospital	0 (0.0)	23 (67.6)	7 (20.6)	4 (11.8)

In-person visits by hospital staff to the school	0 (0.0)	26 (76.5)	5 (14.7)	3 (8.8)


*Note*. Frequency count (*N* = 34).

### Overall Evaluation of HBSP Services

When asked to evaluate the HBSP, over 75% of respondents indicated that they were either *somewhat* or *very satisfied* with their HBSP experience (see Table 7). When asked about more specific aspects of the interaction, the highest proportion of participants *agreed* or *strongly agreed* that HBSP helped to create an individualized re-entry plan for the student and that they communicated well with the educator (both at 61.5%). Open-ended responses also indicated that the most helpful aspects of the HBSP were the overarching communication, support, and recommendations/suggestions for school of record teachers. Common justifications captured via open-ended responses for the overall positive satisfaction scores included communication quality, helping students to keep up with work, or accessible and timely communication. For those respondents who indicated no answer or neutral responses, they most often cited a lack of memory or lack of understanding for the HBSP role.

**Table 7:** Educator Perceptions of Interactions with the [Hospital] School Program.


INTERACTION ITEM	STRONGLY AGREE/AGREE COUNT (%)	NOT SURE COUNT (%)	STRONGLY DISAGREE/DISAGREE COUNT (%)

The [H]SP communicated well with the school regarding the student’s return to school.	14 (53.8%)	8 (30.8%)	4 (15.4%)

The [H]SP communicated well with me regarding the student’s return to school.	16 (61.5)	5 (19.2)	5 (19.2)

The [H]SP provided the school with information related to the student’s academic progress during hospitalization.	14 (53.8)	7 (26.9)	5 (19.2)

The [H]SP helped the student transition back to school following their hospital stay.	13 (50.0)	9 (34.6)	4 (15.4)

The [H]SP helped create an individualized return plan for the student’s return to school.	16 (61.5)	6 (23.1)	4 (15.4)

The individualized return plan considered my/the school’s needs upon return to school.	15 (57.7)	7 (26.9)	4 (15.4)

Overall, I am satisfied with the services the student received from RSP during the inpatient stay	15 (57.7)	9 (34.6)	2 (7.7)

Overall, I am satisfied with the services received from RSP as the student returned to school.	16 (61.5)	8 (30.8)	2 (7.7)


*Note*. Likert Scale ranged from *strongly disagree* to *strongly agree* (*N* = 26).

### Demographic Characteristics and Trends in Responses

**Figure 1:**
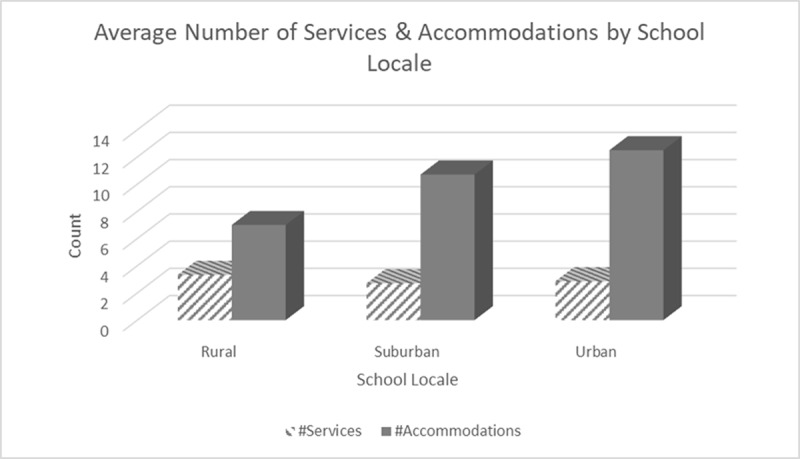
Average Services, Accommodations, and Satisfaction by School Locale.

Further analyses examined the relationships between years’ experience, satisfaction, and number of services or accommodations reported. One statistically significant correlation was found between years of experience and number of accommodations reported (Spearman’s rho = 0.417, *p* = 0.027, *n* = 28). In addition, there was some evidence of different perspectives, services, and accommodations reported based on school locale (see Figure 1), though no statistically significant correlations were found. Unfortunately, our sample size did not allow for additional inferential statistics to be run on these numbers.

When considering respondents’ knowledge and training regarding students’ chronic health conditions, all respondents indicated that they were at least *somewhat* comfortable having students with chronic illnesses in their classroom, despite only 7.89% receiving school-provided education on pediatric chronic illness each year. When asked where they most commonly received their information, respondents most frequently indicated parents, the school nurse, the internet, and/or self-education. Respondents were least likely to indicate receiving information from principals, other teachers/professors, academic journals, or professional newsletters/magazines.

### Open-Ended Responses

Finally, participants completed open-ended response items throughout the survey. One item sought to identify specific challenges students with chronic illness encounter while in school. Challenges respondents indicated were most common from the student perspective included keeping up or getting caught up with material and attendance as well as social isolation and the need for private space. Teachers also mentioned logistical challenges they and/or the school faced upon student return, including transportation, implementing Section 504 plans, developing a routine, and keeping the classroom free from germs.

When asked about potential areas for further improvement, communication was the main concern for respondents. Communication on the specific role or available services from the HBSP, discharge instructions and re-entry plans, as well as the frequency of communication were all noted as important aspects to keep services moving forward smoothly. Some respondents also wondered about potential opportunities for students to join class virtually while still inpatient or on medical homebound.

## Discussion

HBSP services are incredibly important for the continuity of educational opportunities during inpatient hospitalization for CSHCN (Steinke et al., 2016). The present study surveyed private and public school educators about their experiences working with an HBSP to transition students from inpatient hospitalization back to students’ school of record. The survey asked broad questions about the educators’ experiences with the HBSP and the services the HBSP provided during the student’s care transition. In general, the educators’ perspectives of the HBSP were positive; over 60% of the sample were “very satisfied” with the services provided by the HBSP.

Educator perceptions related to communication provided by the HBSP was mixed. When participants were asked to qualitatively share why they were satisfied with the HBSP’s services, many endorsed “communication” from the HBSP to the school. Additionally, communication, support, and recommendations/suggestions for teachers were heavily endorsed as the most helpful aspects of the HBSP’s services. However, 42% of survey respondents were “unsure” if the HBSP checked in on students after they re-integrated into the school setting while 23% endorsed “no.” Perhaps this is connected to the concerns with communication expressed by some of the respondents.

Additionally, qualitative responses to the question asking for suggestions on the improvement of HBSP’s services, communication emerged as a top sentiment. Specifically, educators wanted more information about the role of the HBSP and what services it offers, including what educational programming was provided to the student while hospitalized and more frequent communication from the HBSP, including post-discharge as well as what behaviors may be expected of the student upon re-entry. Meeting this need may prove challenging given restrictions such as the Health Insurance Portability and Accountability Act (HIPAA) of 1996 and the Family Educational Rights and Privacy Act (FERPA) and any additional local policies. These laws and policies restrict the ability of educators and hospital personnel to share information without the express consent of a parent or guardian.

A vast majority of educators in the present study indicated their schools did not provide annual education about pediatric chronic illness. Respondents reported gathering information from a variety of sources (e.g., school nurse, the Internet, parents) to learn more about supporting this population, yet little information was available via structured or formalized training opportunities. This finding is consistent with prior literature indicating that many teachers lack knowledge related to or felt unprepared to work with students with chronic illness due to inadequate training (Hinton & Kirk, 2015; Irwin et al., 2018; West et al., 2013; Wikel & Markelz, 2023). A systematic review unveiled three key areas in which educators are unprepared to support CSHCN: (a) the medical needs of CSHCN; (b) knowledge regarding how medical symptoms and treatments may educationally and cognitively impact a student; and (c) adapting classroom activities to reflect the needs of CSHCN, including managing absences related to the illness and re-entry upon hospitalization (Hinton & Kirk, 2015).

Although most respondents in the current study indicated a general level of comfort in having children with chronic illness in their classroom, a vast majority reported not receiving regular professional development about pediatric chronic illness. Given the complexities of CSHCN, and the prevalence of children with these needs in schools (Wikel & Markelz, 2023), emphasis should be placed on providing educators with more knowledge and training on how to work with this population. Indeed, HBSPs can play an important role in this education (Wikel & Markelz, 2023). For instance, although not the focus of this study, this specific HBSP developed several tools to provide direct education to classroom teachers related to medical needs of students, including “Day-in-the-Life” videos following students during treatment course for select disorders (e.g., cystic fibrosis) and chronic medical condition forms with overview information on the impact of the disease on the student. The HBSP also provided direct education to school personnel virtually or in person as allowed by the school of record and with guardian/parent permission per HIPAA and FERPA regulations. Additional work is needed to examine the impact of these efforts on educator knowledge of CSHCN.

Finally, accommodations and services offered to students upon re-entry by the school of record focused mostly on academic performance and attendance. While both aspects are important—CSHCN may miss a considerable amount of school and fall behind academically (Lum et al., 2017; Marraccini & Pittleman, 2022; Shaw & McCabe, 2008)—schools and HBSPs may miss an important element of re-entry related to supporting student mental health. For instance, the number of students experiencing inpatient hospitalization for psychiatric conditions has significantly increased in recent years (Marraccini et al., 2019), marking an astounding 300% increase in the rate of psychiatric hospitalizations over the last twenty years despite decreasing childhood hospitalizations overall (White et al., 2017). Indeed, children returning from psychiatric hospitalization have endorsed feeling overwhelmed, exhausted, and stressed upon returning to school, and also reported difficulties in accessing school mental health providers (e.g., school counselors) when needed (Marraccini & Pittleman, 2022). Further, data indicate the most common reason for inpatient admission for children ages 10 and older is related to mental health (e.g., depressive disorders, post-traumatic stress disorder; Weiss et al., 2022), yet these children are significantly less likely to receive mental health care (Maternal and Child Health Bureau, 2022).

Adding to the complexity of returning to school upon discharge are the potentially traumatic experiences a child may have experienced due to the illness or injury that initially led to inpatient admission (Kassam-Adams & Butler, 2017). While many children are resilient, some continue to experience medically related traumatic stress for months, or even years, leaving them at increased risk for poorer quality of life and academic outcomes (De Young et al., 2012; Le Brocque et al., 2010). Although in the present study we do not know the specific medical condition related to the student’s hospitalization, we can reasonably infer that at least some students were hospitalized due to mental and/or behavioral health needs and that some had experienced some type of medical traumatic stress. Supporting student mental health is a critical element of reducing longer-term trajectories of posttraumatic stress reactions for children with chronic illness (Le Brocque et al., 2010, 2020) and is an important area of future exploration.

## Implications for Practice

### Communication Between Schools and the HBSP

First, schools’ requests for additional communication from the HBSP to the school is an important element to consider. Communication is an essential feature of successful collaboration between schools and families (Malchar et al., 2020). Indeed, communication is also foundational for creating a unified plan of support for a student returning to school upon reintegration from inpatient hospitalization (Committee on School Health, 2000; Groh et al., 2020; Weiss et al., 2015). Communication should be multidisciplinary (Groh et al., 2020) and triangulated between hospital staff and medical professionals, school staff (i.e., teachers, administrators, school-based mental health personnel), and families (Committee on School Health, 2000). In this domain, advocates support the identification of a school reintegration liaison (SRL) to coordinate communication among all relevant stakeholders (Clemens et al., 2011; Moore et al., 2009).

An SRL plays an important role in supporting cross-system collaboration to facilitate communication among stakeholders in the process of developing a comprehensive reintegration plan for the student (Savina et al., 2014). HBSPs should consider this role and determine who the best person for this would be. Communication plans should consider essential elements such as educational goals (Committee on School Health, 2000) as well as a structured reintegration plan (Marraccini et al., 2019).

### Comprehensive Reintegration Plans

As previously stated, CSHCN face a host of academic, social, socioemotional, and medical needs as they progress through school. As such, comprehensive reintegration plans must capture current level of functioning, goals, and plans to support the student across functioning domains (Marraccini et al., 2019)—in short, they should be individualized for the student and the illness (Marraccini & Pittleman, 2022; Wikel & Markelz, 2023). Such a plan should include a discussion of the individual’s level of functioning, timelines for discharge, and recommendations for the individual upon discharge (Hall & DuBois, 2020). This coordination helps ensure the student’s needs a being met while preventing the duplication of services and minimizing confusion (Hall & DuBois, 2020; Weiss et al., 2015).

HBSPs can coordinate with schools of record to develop plans that include considerations for academics, socioemotional needs, traumatic stress reactions (i.e., traumatic stress reaction after a life-threatening illness, injury, or medical procedure), safety plans, medical needs, environmental considerations (e.g., where classroom materials are stored), interpersonal considerations, and social (re)integration (Le Brocque et al., 2020; Marraccini et al., 2019). Plans should be reviewed and modified as needed (Clemens et al., 2011; Marraccini & Pittleman, 2022).

### Training School of Record Staff

Previous studies have found teachers reported feeling anxious about having students with chronic illness in their classrooms for fear of a medical emergency happening in their room, or not knowing the proper way to respond should such an emergency occur (Hinton & Kirk, 2015; Olson et al., 2004; West et al., 2013). Educators also reported concerns related to how much time and attention would be required to work with and support the student with chronic illness (Olson et al., 2004). HBSP staff can play a critical role in educating teachers about how the child’s illness may manifest in class, such as the academic, socioemotional, and/or cognitive impacts of the child’s illness (Hinton & Kirk, 2015). Indeed, when teachers received factual information related to a child’s medical condition, they reported feeling more confident in being able to support the child in their classroom (Canter & Roberts, 2012).

Relatedly, evidence suggests that teachers are more willing to implement accommodations that they perceive to be less burdensome (West et al., 2013). HBSPs can play an important role in providing evidence-based information related to the student’s medical condition (Wikel & Markelz, 2023) as well as best practices related to student needs, such as academic accommodations, social/interpersonal supports, and environmental considerations. In fact, when trainings have been provided, educators report increased positive attitudes and knowledge related to specific illnesses, and feeling less anxious about having a student with healthcare needs in their classroom (Hinton & Kirk, 2015). There is also some evidence to indicate training can be effectively delivered remotely, and was endorsed as a preferred method by teachers (Brown et al., 2011). Ultimately, development of additional training programs and evaluation of subsequent outcomes is needed (Hinton & Kirk, 2015).

## Limitations and Directions for Future Research

While the current study has many strengths, it is not without limitations. First, the study was limited to an HBSP in one midwestern state in the United States, and sample demographics heavily favored the perspectives of white, female educators and those from urban locales. Due to a high number of incorrect email addresses, the number of potential respondents decreased dramatically from the initial sample. Thus, some analyses were limited due to the sample size despite our response rate (19.6%) being consistent with response rates and associated challenges often seen with web-based surveys (e.g., incorrect email addresses; Daikeler et al., 2020; Nayak & Narayan, 2019). Our sample was comprised of a broad range of school personnel (i.e., educators across six different educational roles), but representation was limited from classroom teachers and school mental health professionals in particular. It is possible the lack of representation of classroom teachers contributed to less accurate reporting of services provided by the HBSP, particularly if peripheral school personnel not directly involved with service delivery responded to the survey.

Future studies should examine HBSP services from these stakeholder groups as classroom teachers are the ones directly seeing the impact of the educational services a student received while hospitalized. Additionally, future research should also consider HBSP services from the perspectives of stakeholder groups such as families, HBSP personnel, and students themselves as well as across school type (e.g., public school, private school, charter school) and level (e.g., elementary school, secondary school). We did not report on the types of chronic illness the students had in relation to the services provided by the HBSP. Future work could examine HBSP services in relation to the type of medical needs of the students being served (e.g., examining the perspectives of educators with students returning from the behavioral health unit). Finally, the study did not examine HBSP services in relation to students’ academic and socioemotional outcomes. Additional research should examine the link between services provided while hospitalized, re-entry plan services and accommodations, and student socioemotional and academic outcomes.

## Conclusion

Children with chronic illnesses returning to school following hospitalization represent an important subset of the population with whom educators interact on a daily basis. HBSP programs are a critical element to supporting CSHCN during hospitalization and are important for the transition of CSHCN returning to school upon discharge.

The current exploratory study provides a broad overview of the services provided by one HBSP to support student re-entry from the perspectives of educators working with those students. Overall, while perceptions related to the HBSP of study were positive, we found perceptions related to communication to be mixed, and a majority of educators reported not receiving professional development related to supporting CSHCN. Additional work is needed to understand the impact of HBSP services on children, families, and the educators that serve them. Toward that end, this study is an important first step in understanding educators’ perspectives on HBSP supports and services.
